# Data-driven dynamical model indicates that the heat shock response in *Chlamydomonas reinhardtii* is tailored to handle natural temperature variation

**DOI:** 10.1098/rsif.2017.0965

**Published:** 2018-05-02

**Authors:** Stefano Magni, Antonella Succurro, Alexander Skupin, Oliver Ebenhöh

**Affiliations:** 1 Institute of Quantitative and Theoretical Biology, Heinrich Heine University, Düsseldorf, Germany; 2 Luxembourg Centre for Systems Biomedicine, University of Luxembourg, Esch-sur-Alzette, Luxembourg; 3 Botanical Institute, University of Cologne, Cologne, Germany; 4 University California San Diego, La Jolla, CA, USA; 5 Cluster of Excellence on Plant Sciences (CEPLAS), Düsseldorf, Germany

**Keywords:** heat shock response, dynamical modelling, *Chlamydomonasreinhardtii*, heat shock proteins, natural temperature variation, mathematical model

## Abstract

Global warming exposes plants to severe heat stress, with consequent crop yield reduction. Organisms exposed to high temperature stresses typically protect themselves with a heat shock response (HSR), where accumulation of unfolded proteins initiates the synthesis of heat shock proteins through the heat shock transcription factor HSF1. While the molecular mechanisms are qualitatively well characterized, our quantitative understanding of the underlying dynamics is still very limited. Here, we study the dynamics of HSR in the photosynthetic model organism *Chlamydomonas reinhardtii* with a data-driven mathematical model of HSR. We based our dynamical model mostly on mass action kinetics, with a few nonlinear terms. The model was parametrized and validated by several independent datasets obtained from the literature. We demonstrate that HSR quantitatively and significantly differs if an increase in temperature of the same magnitude occurs abruptly, as often applied under laboratory conditions, or gradually, which would rather be expected under natural conditions. In contrast to rapid temperature increases, under gradual changes only negligible amounts of misfolded proteins accumulate, indicating that the HSR of *C. reinhardtii* efficiently avoids the accumulation of misfolded proteins under conditions most likely to prevail in nature. The mathematical model we developed is a flexible tool to simulate the HSR to different conditions and complements the current experimental approaches.

## 1. Introduction

As a consequence of global warming, plants are more and more subject to heat stress, a condition that can severely reduce crop yield [1,2]. Understanding how plants react to such a stress is of crucial importance in developing metabolic engineering approaches or treatments to improve crop plant resistance to heat. In general, when exposed to increased temperature organisms react with a heat shock response (HSR), which to a certain degree allows adaptation to the new condition. However, while the HSR has been considerably investigated experimentally, the complementary theoretical activities have been rather limited. In this work, we seek to remove this gap by developing a mathematical model of the HSR in the photosynthetic model organism *Chlamydomonas reinhardtii* and investigate, in particular, the response dynamics under different experimental conditions. By this mathematical approach to the HSR in *C. reinhardtii*, we show that at the quantitative level, the HSR differs substantially if an increase in temperature of the same magnitude occurs rapidly, as often applied in typical laboratory heat shock experiments, or gradually, as expected in most natural environments.

The green microalgae *C. reinhardtii* is a widely studied, easy to grow photosynthetic model organism with promising industrial applications like biopharmaceuticals, biofuels and hydrogen. Thus motivated, different aspects of the HSR in *C. reinhardtii* have been experimentally investigated, as reviewed, e.g. by Schroda *et al.* [3].

In both the land plant *Arabidopsis thaliana* [4,5] and the green alga *C. reinhardtii* [6], the HSR is generally triggered by a heat-induced accumulation of mis- or unfolded proteins and leads to the activation of a heat shock transcription factor (HSF) through a series of sensor and signalling events. The HSF, in turn, promotes the expression of heat shock protein (HSP) genes, and subsequently leads to the synthesis of proteins, some of which act as chaperones responsible for refolding the degenerated proteins back to their correct three-dimensional structure [7]. The precise temperature at which the denatured proteins accumulate depends on the typical temperature range in which an organism grows [8]. For *C. reinhardtii*, it has been shown that a temperature of *T*_0_ = 36°C is sufficient to detect a HSR [9].

To investigate systematically the HSR dynamics and the underlying mechanisms, we developed a mathematical model based on available experimental data. The construction of a mathematical model itself often provides a high degree of insight, because based on the essential features of the system under investigation it identifies the key components responsible for the characteristic system properties [10,11]. It thus allows discriminating between important and less important entities and provides a powerful technique to verify whether our general understanding of a system is basically correct and whether the interacting molecular mechanisms that have been identified experimentally are sufficient to reproduce and thus explain observed behaviours.

One of the earliest theoretical studies of the eukaryotic HSR considered mainly the influence of misfolded proteins and did not include a detailed description of transcriptional regulation [12]. Modelling of the transcriptional regulation was firstly used to study prokaryotic systems, in particular *E. coli*. This was done by Srivasta *et al.* [13] employing a stochastic approach, and by Kurata *et al.* [14] with a deterministic model. More recently, Rieger *et al.* [15] proposed a mathematical model to describe the HSR in HeLa cells with a detailed model of nuclear events. A model of the thermal adaptation in *Candida albicans*, a fungal pathogen of humans, focuses on the auto-regulatory mechanism involving HSF1 and HSP90 but does not include a detailed description of transcriptional regulation [16,17]. The modelling of the multi-scale heat stress response in the budding yeast *Saccharomyces cerevisiae* is discussed by Fonseca *et al.* [18], and Sivery *et al.* [19] studied the role of HSF1 during the HSR in mammals.

Here, we develop a data-driven mathematical model for the HSR in the green algae *C. reinhardtii* with the main purpose of (i) verifying whether our understanding of the mechanisms of HSR are not only qualitatively but also quantitatively consistent with experimental observations, and (ii) providing a generic theoretical framework, by which new predictions can be made (such as responses to chemical treatments or genetic modifications) and thus novel hypotheses can be generated. For this purpose, we first introduce the considered HSR signalling network used to implement the mathematical description and characterize the typical behaviour based on parameterization by data from the literature. We then validate the model by simulations of independent experimental data including experiments with specific inhibitors and ‘double heat shock’ experiments. Finally, we employ the model to simulate interesting conditions that have not yet been tested experimentally, and we demonstrate the predictive power of the model and its usefulness in providing a fundamental understanding of the system's dynamics.

## 2. Mathematical model

### 2.1. Model description

The design of the model is based on the underlying signalling network inferred from experimental findings [6]. In *C. reinhardtii*, the only HSF (among the two encoded in the genome) known to be activated by heat shock is HSF1 [20], which initiates the synthesis of HSPs [21]. HSP70A and HSP90A are the predominant cytosolic chaperon complexes and HSP70B and HSP90C the analogues in the chloroplast, respectively [22]. As our model aims at a general description of the HSR, we describe HSF1 as HSF in the model and only consider one generic HSP (HP) which represents any HSP present in *C. reinhardtii*.

**Figure 1.**
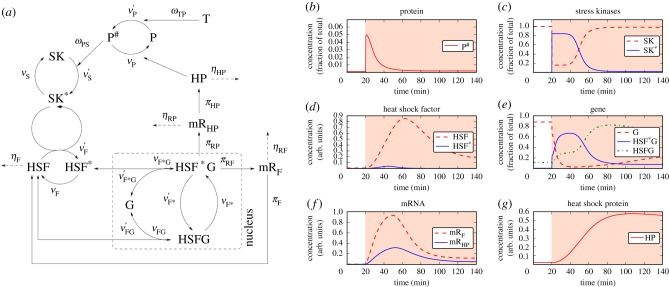
The dynamical model of the HSR. (*a*) Scheme of the signalling network used to model the HSR. This scheme is extracted from the experiments performed by Schmollinger *et al.* [6] and inspired by the signalling mechanisms hypothesized in fig. 11 therein, to which we add gene transcription and apply some simplifications as explained in the text. This signalling network represents the base to build our mathematical model of the HSR described in detail in electronic supplementary material, §A. Temperature T acts via the Arrhenius Law *ω*_TP_ on the proteins P. Higher temperature increases *ν*′_P_ leading to more degenerated proteins P^#^. This activates stress kinases SK (SK* when active) by a Hill kinetics *ω*_PS_ which increases phosphorylation of the heat shock factor HSF (HSF* when active). If HSF* is bound to the gene G, mRNA for the heat shock factor HSF and for the heat shock protein HP is generated by the corresponding production rates *π*, respectively, indicated by mR_HP_ and mR_F_. The mRNA is translated into the proteins HP and HSF and degraded by rates *η*. HSF*G indicates *HSF* active and bound to gene, *HSFG* indicates *HSF* inactive and bound to gene. (*b*–*g*) Typical behaviour of the model illustrated inducing a HSR via an increase of the temperature from 25°C to 42°C applied at *t* = 20 min (represented by a red background in the figures). (*b*) Owing to temperature increase at *t* = 20 min functional proteins P are misfolded leading to an increased P^#^ level. (*c*) The degenerated proteins bring inactive stress kinases SK into their active form SK*. (*d*) Due to active stress kinases, the heat shock factor (HSF) is phosphorylated (HSF*). (*e*) The heat shock factor HSF binds to free gene loci G, the bound form HSF*G activates mRNA production, and HSF un-binding blocks transcription. (*f*) The initiated gene transcription leads to mRNA production of the HSF and the heat shock protein as shown. (*g*) Owing to translation of the corresponding mRNA, the HP concentration increases until the response is switched of. The small degeneration rate of the chaperon leads to a slow decrease after the onset of the HSR. The normalization factors used to represent the concentrations in arbitrary units can be found in electronic supplementary material, table S3. (Online version in colour.)

Schmollinger *et al.* [6] performed a series of experiments which led to a hypothesized signalling network (depicted in fig. 11 in [6]). From these results, we derive the signalling network schematically depicted in figure 1*a* by performing the above simplifications and detailing the transcription of the HSF and HSP genes. This signalling network represents the base for building our mathematical model of the HSR. In accordance with experimental evidence, a temperature increase triggers the HSR by the accumulation of degenerated proteins P^#^. Their presence activates a stress kinase (SK), which in the active form SK* phosphorylates the heat shock factor HSF. The phosphorylated (HSF*) and unphosphorylated (HSF) heat shock factor can bind to the transcription factor binding sites of various genes, coding for key proteins involved in the HSR, including HSF itself and HSPs (HPs). In the model, the amount of all these genes is described by one generic variable G, and the transcription of different mRNAs is represented by the individual transcription rates *π*. Binding of the active form HSF* to the transcription site of genes G induces the production of mRNA coding for HSP (mR_HP_) and for the heat shock factor itself (mR_F_), whereas the inactive form HSF blocks the transcription. The mRNAs are subsequently translated into the corresponding proteins HP and HSF (with rates *π*_*HP*_ and *π*_*F*_), respectively. The increase in HSF concentration leads to a higher occupation of the corresponding gene transcription site with the inactive form. The increased concentration of chaperones HP increases the repair of the degenerated protein state P^#^ to their functional form P until a new steady state is reached.

The full model is described by 12 dynamic variables (electronic supplementary material, table S1), each representing the concentration of the corresponding network component. The dynamics of these variables are governed by a set of ordinary differential equations given in electronic supplementary material, table S2, that follow from the considered reactions and corresponding rate expressions given in electronic supplementary material, table S5, where *ω* describe regulatory processes, *ν* activation and deactivation steps, *π* synthesis rates and *η* degradation rates.

For the majority of these rate laws, we assume mass action kinetics and describe regulatory processes *ω* by nonlinear dependencies. The effect of the temperature on the denaturation (unfolding or misfolding) of proteins is described by means of the Arrhenius Law, with an activation energy in the range reported in the literature [23,24]. The activation (phosphorylation) of the SKs given by *ω*_PS_ obeys a Hill kinetics. Furthermore, the action of the phosphorylated SK*, the enzyme phosphorylating HSF, is described by a Michaelis–Menten behaviour as expected for typical enzymatic kinetics. A detailed description of the mathematical model can be found in electronic supplementary material, §A.

### 2.2. Typical behaviour of the model

To investigate the typical behaviour of the model, we simulate a heat shock at time *t* = 20 min by instantaneously increasing the temperature from 25°C to 42°C. The inferred model parameters are explained below. This scenario mimics a standard experimental design, in which the temperature is rapidly increased to induce a HSR. The time evolution of the concentrations of the molecular species described by the model is depicted in figure 1*b*–*g* where a red background indicates the period during which a heat shock temperature is applied. These concentrations are normalized to a reference value for each panel, as summarized in electronic supplementary material, table S3. The quantities of figure 1*b*,*c*,*e* are normalized to the three conserved quantities representing, respectively, the total amount of [P] + [P^#^], [SK] + [SK*] and [G] + [HSF *G] + [HSFG], thus the vertical scale represents the fraction over the total, allowing for direct comparisons with relative values from experiments. Quantities in figure 1*d*,*f*,*g* are expressed in arbitrary units.

Owing to the temperature increase, the functional proteins become misfolded (concentration in figure 1*b*). The concentration of the functional form P suddenly decreases due to the nonlinear *ω*_TP_ relation and the misfolded form P^#^ increases correspondingly. The latter form induces the transition of the SKs into their active form SK* (figure 1*c*). SK* phosphorylates the HSF (figure 1*d*). The activated HSF* activates gene transcription (figure 1*e*). The amount of free gene G decreases and the active form with phosphorylated HSF bound, HSF*G, increases rapidly. Simultaneously, the gene bound to the inactive form of HSF (HSFG) increases as well, but with a slower dynamics. The activated gene induces mRNA production (figure 1*f*) for both HSF and HSP. These mRNAs are translated, leading to an increase of the HSF itself (figure 1*d*) and of the HSP (figure 1*g*). The increased chaperon level (figure 1*g*) leads to re-folding of degenerated proteins into their functional form (figure 1*b*), which eventually leads to a termination of the response. This analysis illustrates that the model is able to realistically describe the HSR.

### 2.3. Model parameterization

**Figure 2.**
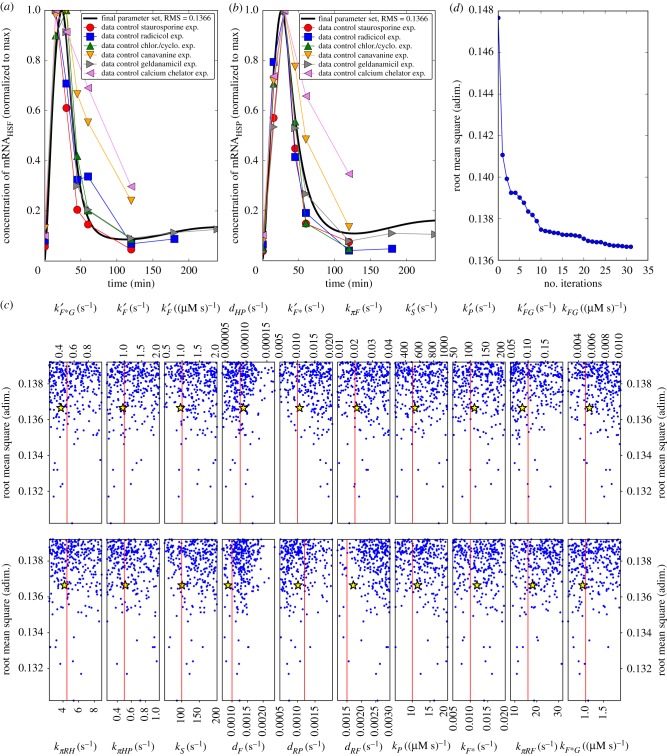
Model parameterization. (*a*,*b*) Data from the controls of the feeding experiments of Schmollinger *et al.* [6]. These correspond to six curves representing the time evolution of the concentration of mRNA coding for HSF1 (*a*), and six curves for the mRNA coding for HSP90A (*b*), under heat shock and no inhibitor treatment. The superposed continuous thick black line shows the model prediction obtained with the final parameter set. (*c*) Projection w.r.t. each of the 20 parameters of the Monte Carlo (MC) scan of the parameter space, for the points corresponding to the 300 random parameter sets with lowest RMS among the 10^5^ sets randomly generated. The RMS corresponding to these 10^5^ sets ranged between 0.130 and 0.700, while this panel is strongly magnified and only shows parameter sets with RMS between 0.130 and 0.139. The vertical line in each sub-panel indicates the fiducial value of the corresponding parameter, i.e. the starting point of the two alternative calibration approaches used, the MC described in this panel, and the gradient search eventually employed. The yellow star in each sub-panel indicates the parameter values finally retained after the gradient search, referred to as the ‘final parameter set’ (electronic supplementary material, table S4). These values are relatively close to the fiducial values, and the corresponding RMS of 0.137 is close to the lower extreme of the range of values obtained with the MC (0.130 to 0.700). (*d*) RMS decrease for subsequent iterations of the gradient search algorithm. (Online version in colour.)

A general challenge in modelling biological systems is the identification of system parameters because often these are not directly accessible experimentally. None of the rate constants listed in electronic supplementary material, table S4 are explicitly known. Still for some of them a reasonable range can be estimated from databases [25]. In particular, we ensured that the rate constants *k*_*P*_, *k*_*FG*_ and *k*_F*__G_ were kept in a range lower than the diffusion controlled limit of enzymatic reaction rates estimated in [26, tables 6–8]. We further select a value for *k*_F*__G_ with the same order of magnitude of the activator association rate reported in [27]. Considering this information, we first manually tuned all the parameters to reproduce the qualitative behaviour of the experimental data. These parameters are referred to as the ‘fiducial parameter set’, reported in the second column of electronic supplementary material, table S4. We then used this fiducial parameter set as a starting point for a deeper investigation of the parameter space represented by the 20 rate constants. For this, we divided the experimental data available from the literature in two groups, one used to calibrate the model and the other to validate the model. The data used for calibration comprise the controls of feeding experiments performed in Schmollinger *et al.* [6] including six time course curves of HSF1 mRNA concentration, and six curves for the mRNA coding for HSP90A, under heat shock and no inhibitor treatment. These data are shown in figure 2*a*,*b*. We have then defined an objective function reflecting the quality of the fit by a root mean square (RMS) of the deviations between model simulations and experimental data. We first performed a Monte Carlo (MC) scan of the parameter space to gain insight into its structure and then a gradient search to find a set of parameters which locally optimizes the objective function.

To perform an MC exploration of the parameter space, we assumed a flat prior probability distribution between half and two times of the fiducial value of each parameter. Then, by randomly extracting a value for each parameter from these distributions, we generated 10^5^ randomized parameters sets. For each set, we computed the corresponding value of the RMS with respect to the data of the first group. We obtained values of the RMS ranging approximately from 0.13 to 0.70. The fiducial parameter set has a RMS w.r.t. the controls of the feeding experiments of 0.147, which is already remarkably close to the lowest values obtained for the random parameter sets. For illustration, we select the best 300 parameter sets, corresponding to the lowest values of the RMS, and we show in figure 2*c* (for each of the 20 parameters separately) the values of the RMS versus the value of each parameter.

We observe that for the vast majority of the parameters no preferred interval in which the lowest values of the RMS occur more often can be identified. This observation means that many different configurations in the parameter space would allow us to obtain a small RMS with respect to the data. It is thus important to note that the calibration data we used, which only provide a relative quantification of two molecular species, namely *mR*_*F*_ and *mR*_HP_, are not sufficient to identify the model parameters uniquely, nor to provide estimates of these parameters independently of the other parameter values. To investigate these correlations further, in electronic supplementary material, §B, we also investigate pair-wise relationships among model parameters (see electronic supplementary material, §B and figure S5). The limited available data for model calibration stresses the importance of an accurate model structure, which here was constructed based on the experimental findings in [6].

Thus, having shown via a global random scan of the parameter space that almost no region of the parameter space explored is preferred by the RMS, we decided on a local optimization procedure, thus performing a gradient search starting from the point in the parameter space represented by the fiducial set of parameters, employing the steepest descent method. As shown in figure 2*d*, our algorithm stopped after several iterations and returned the set of parameters listed in the third column of electronic supplementary material, table S4 as final values. The corresponding value of the RMS w.r.t. the controls of the feeding experiments is 0.137. This value lies very close to the lower boundary of the range obtained with randomized sets (0.131 to 0.700). We prefer it over randomized sets with a slightly lower RMS because it is closer to the manually tuned set of parameters which already performed well, and it should lie very close to a local minimum (thus RMS should only increase if we perturb the parameters). We accept this set as the ‘final parameter set’ to perform all subsequent model analyses, because it adequately reflects the limited experimental data available, and with these parameters the model exhibits the essential features of the heat shock system, which include a rapid initial response followed by the production of HSPs leading to the removal of misfolded proteins. A more detailed description of the calibration is provided in electronic supplementary material, §B. Therein, we also perform a sensitivity analysis to assess how small variations in each of the 20 rate constants influence the RMS (electronic supplementary material, figure S6).

## 3. Results and discussion

### 3.1. Model validation by experimental data

After parameterization, we used the model with its inferred parameters to quantitatively simulate the dynamics of several independent key experiments to validate the model and investigate underlying dynamical properties. We first simulated the feeding experiments performed in Schmollinger *et al.* [6] (§3.1.1), where specific inhibitors have been applied in different concentrations and the effect on the HSR was measured. Subsequently, we simulated the double heat shock experiments performed in Schroda *et al.* [28], where an additional heat shock was applied to quantify the minimum relaxation time needed to observe a full second response (§3.1.2). Finally, we tested the model against measured HP concentrations [29] (§3.1.3).

#### 3.1.1. Inhibitor treatments

In the systematic experiments reported in Schmollinger *et al.* [6], *C. reinhardtii* cells have been fed with different concentrations of specific inhibitors, and the effect on the HSR has been observed by monitoring the temporal evolution of mRNA concentrations of mainly the HSF1 and HSP90 genes. We specifically consider the two inhibitors staurosporine, a protein kinase inhibitor [30], and radicicol, a specific inhibitor of HSP90 [31]. We simulate these experiments by altering the corresponding rate constants to mimic the effect of the inhibitors, and apply the same heat shock conditions as in the experiments, simulating a sudden temperature increase from 25°C to 40°C at 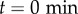.

##### 3.1.1.1. Staurosporine

**Figure 3.**
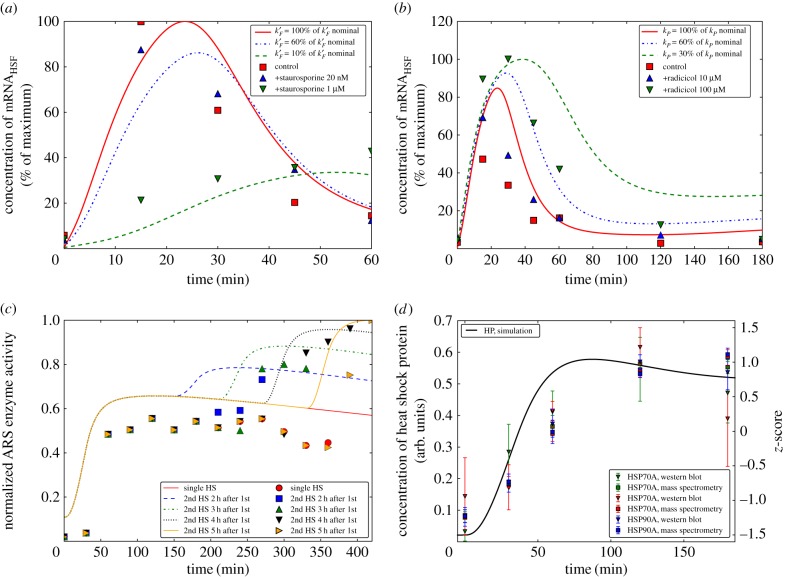
Comparisons between model predictions and data. Feeding experiments by Schmollinger *et al.* [6] using staurosporine (*a*) and radicicol (*b*). (*c*) Simulating the double HS experiment and comparison with the corresponding data from Schroda *et al.* [28]. As in that experimental study, two HSs of 30 min duration each were applied to our model with an interval of 2, 3, 4 and 5 h (blue, green, black and yellow curve, respectively), and compared to the response to only one HS of 30 min duration (solid line without second increase). We see that a full HSR, in which the increase in the concentration of *HSP* (and thus in the ARS enzyme activity) has approximately the same magnitude as for the first HS, is possible only when the second HS occurs at least 5 h after the first HS. (*d*) Comparison between the model predictions for the variation of the concentration of HP under HS and the corresponding data from Mühlhaus *et al.* [29]. The scale on the left side refers to the simulation results, the scale on the right side to the data. An exact correspondence of the two scales is not possible because we lack the necessary information. (Online version in colour.)

Staurosporine is a protein kinase inhibitor. We, therefore, simulate the effect of applying staurosporine by lowering the rate constant *k*′_*F*_, which determines the reaction rate *ν*′_*F*_, by which SK activates the HSF. The simulation results are shown in figure 3*a*, together with redrawn experimental data from fig. 1B of Schmollinger *et al.* [6].

The simulations clearly exhibit a reduced maximal HSF mRNA concentration and a delayed response in a dose-dependent manner which is in accordance with the experimental data (figure 3*a*). Because the experimental data are normalized to the maximal response, a direct comparison of the simulated concentrations is not possible and the applied staurosporine concentration cannot directly be translated into a reduced rate constant. However, the simulated responses for *k*′_*F*_ at its nominal value of 1.09 s^−1^ and for values reduced to 60% and 10% of that value led to a remarkable agreement between simulation and experiment, in which staurosporine was applied in concentrations of 20 nM and 1 mM, respectively. Not only is the qualitative behaviour well captured but also the timing of the response as well as the relative reduction of the mRNA signal is quantitatively reproduced.

##### 3.1.1.2. Radicicol

Radicicol is a specific inhibitor of HSP90 activity. Therefore, we simulate the effect of radicicol by lowering the rate constant *k*_*P*_, which determines the reaction rate *ν*_*P*_, by which the HSP refold the unfolded proteins P^#^ back to their functional form. The simulation results and the corresponding data (reproduced from fig. 4B of Schmollinger *et al.* [6]) are displayed in figure 3*b* for three values of the rate constant *k*_*P*_ corresponding to the reference value of 9.938 (mM s)^−1^, and for a reduction to 60% and 30% of that value. We see that decreasing the rate constant results in an increased amplitude and delayed attenuation of the HSR. The data from Schmollinger *et al.* [6] shown in the same panel for a control and radicicol concentrations of 10 and 100 μM demonstrate that a similar behaviour is observed in the experiments. Interestingly, the magnitude of the responses are qualitatively reproduced by our model and appear much more pronounced in the experiment.

#### 3.1.2. Double heat shock

In Schroda *et al.* [28], the ARS gene that encodes for the enzyme arylsulfatase was placed under control of the HSP70A promoter. The study demonstrated that whenever the HSP70A gene is activated also the ARS enzyme is produced. Under the assumption of a direct proportionality between the concentration of the ARS enzyme and its activity, the authors could monitor the activity of the HSP70A promoter by measuring the ARS activity. This construct was then used to systematically expose *C. reinhardtii* cells to two subsequent heat shocks to determine the minimum time the cell needs to observe a full HSR for the second heat shock. It turned out that the heat shock system relaxes within approximately 5 h to its initial state.

To compare the experimental results to our model simulations, we extended the model accordingly, also including transcription and translation of the ARS enzyme (for details see electronic supplementary material, §C.2, where we also provide the corresponding equations and parameters values). In figure 3*c*, we display the simulation results and the corresponding experimental data redrawn from fig. 7b of Schroda *et al.* [28], where two heat shocks of 30 min duration were applied with the intervals of 2, 3, 4 and 5 h, respectively. It can clearly be observed that both in simulation and experiment the second HSR increases in intensity with increasing time between the treatments, eventually reaching its full activity after approximately 5 h. Again, the model results are in good qualitative agreement with the experimental data, but clear quantitative deviations can be observed. For example, the simulations systematically display an earlier response to the heat shocks (first and second) than the experimental data. While the exact reason for this is not known, it is striking that also the experimental response to the first heat shock occurs later than in the control experiments used to calibrate the parameter sets (figure 2*a*,*b*). A possible explanation for these discrepancies is a considerable time lag introduced by the transcription and translation of ARS. Further, it must be considered that the double heat shock experiment was performed with a transformed line, and potentially the behaviour deviates slightly from the wild-type. However, the qualitative agreement between simulation and data provides a further validation of our model. Most important, it also illustrates the flexibility of our model, which as we have shown can easily be extended to include further reactions. This is of particular relevance in view of its possible applications, e.g. to characterize the production of any protein whose corresponding gene has been put under the control of temperature by means of fusion with a HSP promoter.

An interesting observation when analysing the model simulations is that even after 5 h, the concentration of HSP does not yet relax to its initial value before the first heat shock. While the second heat shock leads to misfolded proteins and triggers an almost full HSR in terms of the observed HSP70 promoter activity, the amount of misfolded proteins is dramatically lower than during the first heat shock (see electronic supplementary material, figure S8 and §C.1). This indicates that the production of HSF resulting from the first HSR and the accumulation and slow degradation of HSP have the role of preparing the organism for future stress situations similar to those encountered in the past. Thus, the slow turnover of HSP may implement a short-term molecular memory of experienced heat stress, similar to the observed short-term memory of previously experienced light stress recently discussed and described by a mathematical model in Matuszyńska *et al.* [32].

The model simulations allow for a novel interpretation of the experimental double heat shock results. While the activity of the HSP70 genes seemingly indicates a full HSR, our simulations suggest that the first and second response differ quite fundamentally. During the first exposure, HSP needs to be synthesized de novo from practically zero concentration and, therefore, the corrective response to refold the denatured proteins is slow, whereas in the second heat shock after 5 h the remaining HSP level is still sufficient to rapidly counteract the temperature-induced denaturing of proteins and the total level of misfolded proteins remains very low. However, even this low level is sufficient to induce expression of the HSP genes, so that the mRNA level during the second response is as high as during the first.

#### 3.1.3. HP expression

Unlike the study of the feeding experiments, where model predictions and experimental data were compared at the level of mRNA production, we next compared our simulations with time course data of the HP concentration [29]. To simulate the experiment we apply at 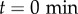 a heat shock by increasing the temperature from 25°C to 42°C. We compared the model simulated behaviour of HP with the experimental data on HSP70A, HSP70B and HSP90.

Figure 3*d* shows that the model reproduces well the qualitative behaviour of the data. Although the model predicts a slightly faster increase in HP concentration during the first 100 min than experimentally observed, it reproduces the key feature that increase is initially slow, then accelerates, while at later times the HP concentration again slightly decreases. Quantitatively, experimental data are provided in terms of the *z*-score, measuring the distance of single data points from the mean in terms of standard deviations. As the information needed to convert this scale to concentrations is not available, we superpose our simulation results and the data, and indicate the two different scales of the *y*-axes.

### 3.2. Modelling natural temperature variation

As demonstrated above, our mathematical model, which has been calibrated to control experiments only, can reproduce drug treatments, the double heat shock experiments and the data on HP abundance reasonably well. The agreement of simulation results and experimental data therefore supports the notion that our current understanding of the HSR is basically correct. Our model can therefore serve as a theoretical framework in which data can be interpreted in a quantitative way. Another purpose of model building is the ability to make novel predictions. We have therefore employed our model to simulate scenarios that provide insight into our understanding of the HSR, but which are either difficult to test or have not yet been tested experimentally, as further exemplified in electronic supplementary material, §D.

**Figure 4.**
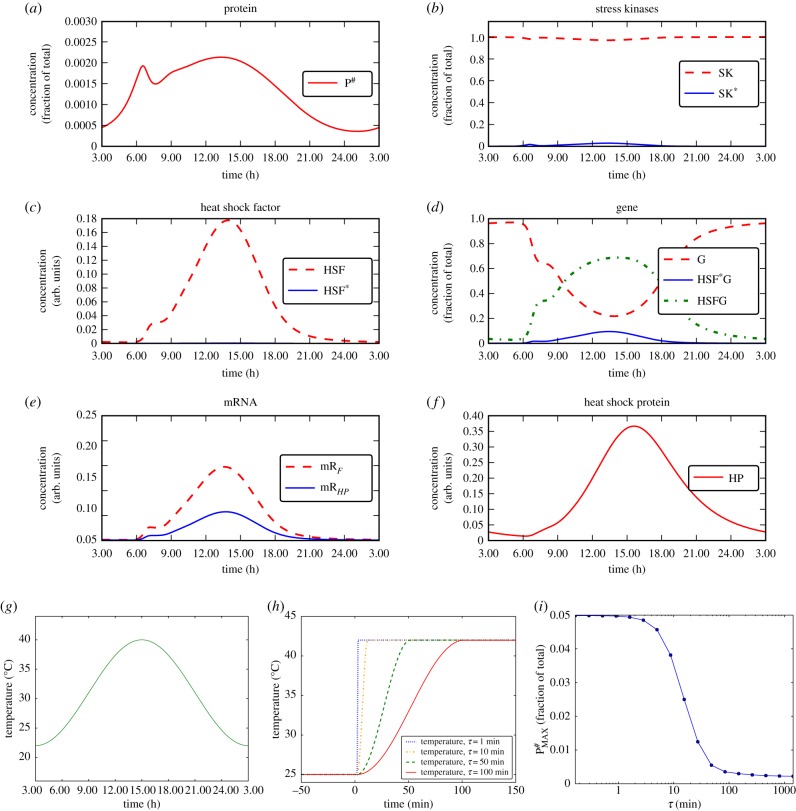
HSR is tailored to handle natural daily temperature variation. (*a*–*f*) Simulation of the response of the system to a temperature fluctuation approximating a typical hot day, shown in (*g*). The concentration of unfolded proteins is kept very low, well below 1% of the total amount of proteins [P] + [P^#^] (note the different vertical scale in (*a*), compared with previous figures). (*h*) We further consider temperature variations from 25°C to 42°C occurring across increasing periods of time, here shown from 1 to 100 min. (*i*) Study of how the maximum concentration of unfolded proteins accumulated at steady state during a heat shock depends on how fast the temperature has increased from the initial (lower) value to the final (higher) value, considering times from 10^−1^ to 10^3^ min. (Online version in colour.)

To reproduce the typical experimental set-up employed in many studies, we have so far considered step-wise temperature increases as heat shocks throughout this work. However, these situations do not reflect the natural environment. Hence, what kind of heat shock is a *C. reinhardtii* cell going to experience in the wild? This green algae is widely distributed around the world in various environments such as soil and fresh water. Thus, a natural heat shock condition it encounters is the daily variation of the temperature, which is low at night, increases during the day, reaches a peak and then drops again. To investigate the natural HSR in *C. reinhardtii*, we simulate an idealized temperature variation mimicking a hot day by imposing a sinusoidal variation between 22°C and 40°C with a period of 1 day and a maximum at 15.00 (figure 4*g*).

As we can see from figure 4*a*–*f* , the concentrations of the mRNAs (figure 4*e*) and of the HSF (figure 4*c*) have a steep increase, which leads to a maximum and then a much slower decrease. In particular, in figure 4*a,* we can appreciate the peak in the concentration of degenerated proteins at approximately 06.00. This dynamic is due to the activation of the SK, which follows a Hill kinetics. It is this HSR which lowers the concentration [P^#^] after 06.00. The response remains until the evening and during this time the accumulation of P^#^ due to the increase in temperature is counterbalanced by the HSR (this balance originates the second peak in figure 4*a*). When the temperature becomes sufficiently low in the evening the level of unfolded proteins P^#^ decreases considerably, thus there is no need of a HSR until the next day.

Importantly, the accumulation of unfolded proteins remains considerably smaller than 0.25% of the total amount of proteins ([P] + [P^#^], figure 4*a*). This is remarkable because this value is more than one order of magnitude smaller than that obtained for a stepwise increase in the temperature, figure 1*b*. As unfolded proteins are undesired by the cell, it is meaningful that the HSR, which for a sudden increase of 20°C is not fast enough and allows for a certain transient accumulation of unfolded proteins, is on the other hand perfectly capable of preventing the accumulation of unfolded proteins during a heat shock like those that occur in nature.

The circadian clock of *C. reinhardtii* is well studied [33,34] and it is known to regulate also some HSP as e.g. HSP70B, which exhibits a maximal concentration at dawn. The HSR that we model is not a circadian clock, and has no oscillatory behaviour on its own. We, nevertheless, observe from figure 4*b*–*f* that the HSR is activated just before dawn, i.e. at approximately 06.00, when the concentration of P^#^ shown in figure 4*a* becomes sufficiently high to trigger the HSR. Then, the intensity of the HSR reaches a maximum approximately 1.5 h before the maximum of the temperature, which occurs at 15.00. It then drops slowly during the rest of the day, it is largely absent when night comes, and it remains off over night. We observe in figure 4*a*–*f* that, as soon as the temperature increase has led to a sufficient accumulation of P^#^ to trigger a HSR, this response is strong enough not only to handle the currently present amount of P^#^, but also to prevent the subsequent temperature increase (which still continues for hours) to lead to an accumulation of [P^#^] above the level which triggered the initiation of the HSR. This allows the cell to remain exposed to high temperatures for several hours during the day accumulating far less unfolded proteins than when the temperature increase occurs within minutes instead of hours. This observation allows us to speculate and propose the hypothesis that the HSR might be adapted to naturally occurring, smooth daily temperature variations rather than abrupt temperature changes. The system's parameters could thus be the result of adaptation to variable thermal environments, such that the HSR would be well suited to handle daily temperature fluctuations.

To investigate how the maximum concentration of unfolded proteins accumulated during a heat shock depends on the dynamics of the temperature increase from the initial value *T*_low_ to the final value *T*_high_, we systematically simulated heat shocks in which the temperature raised from *T*_low_ = 25°C to *T*_high_ = 42°C in a cosinusoidal way during a time *τ* and subsequently stayed at *T*_high_ (figure 4*h*). We screened for various values of *τ* as shown in figure 4*i*. We observed that for an instantaneous increase in temperature, up to increases which require approximately 1 min (similar to those provided to cells during experiments), the maximum value of the degenerated protein concentration does not change, and exhibits a very large value. For *τ* between approximately 1 and 100 min there is a steep fall, and another plateau is reached for *τ* larger than approximately 100 min. These are the timescales which a *Chlamydomonas* cell is more likely to experience for an increase of temperature in natural conditions. The accumulated concentration of HSP is much lower than for shorter times *τ*, contributing to generate the hypothesis that the HSR dynamics might be adapted to natural temperature variations (note that the scale on the horizontal axis of figure 4*i* is logarithmic). In electronic supplementary material, §E, we show that these results are robust against changes in the parameters *m* and *P*_0_ of the Hill kinetics term *ω*_*PS*_ by which SK gets activated (electronic supplementary material, table S5).

One of the main advantages of a mathematical model, inferred and calibrated from experiments, is that it allows situations to be simulated that are difficult to test with experiments and enables computation of quantities that are difficult to measure. To outline the potential of our model, we employed it to simulate systematically further situations. We have been able, as described in electronic supplementary material, §D.1, to show the ability of the system to acclimate to temperatures higher than usual during heat shocks longer than 3 h by shifting to a new steady state. Two distinct phases are clearly visible in electronic supplementary material, figure S9*a*–*f*: an early heat shock lasting for about the first 3 h, and a late heat shock in which the system shows acclimation (a new steady state is reached), compatible with the experimental findings of [35]. We have subsequently studied in electronic supplementary material, §D.2 how the steady-state concentrations depend on the temperature at which the steady state is reached. As shown in electronic supplementary material, figure S9*g*–*l*, for not too high temperatures the concentration of unfolded proteins [P^#^] is kept very close to zero. On the other hand, for too high temperatures the HSR cannot prevent the accumulation of degenerated proteins and the cell dies. We have finally been able to systematically investigate how the accumulation of HSPs depends on the combination of temperature and duration of the heat shock in electronic supplementary material, §D.3, which we illustrate in electronic supplementary material, figure S9M.

## 4. Conclusion

In this work, we have developed a data-driven mathematical model for the HSR in *C. reinhardtii*, a photosynthetic model organism. We have extracted the signalling network structure from various experiments, and experimental data are used for model parameterization.

The model is based on a number of simplifying assumptions. Foremost, we assume that the simulated cells are close to a stationary state, in which the total numbers of proteins remain constant. This, however, may be inappropriate if either extreme and prolonged stress conditions apply, or if synchronized cells in periodic diurnal conditions are considered. Especially the latter is important for our predictive study regarding the response to natural daily temperature variations. It is well known that HSPs also underlie a circadian rhythm. For a future study, it would therefore be interesting to link the gene expression dynamics with the output of a model of the circadian clock, in order to study how the results obtained here would be influenced by including dynamic protein concentrations. Such an extended model would moreover provide the possibility to investigate and predict how the HSR depends on the time of the day when it is applied.

For model validation, we have tested the model's behaviour with independent experimental results extracted from the literature; the following were not employed for the calibration: feeding experiments, double heat shock experiments and HP expression. By this, we have shown that the model is able to reproduce very well the main features of various experimental datasets. This capability shows that our conclusion about the signalling mechanism is plausible and robust. Even though the model is based on simplifying assumptions, it exhibits very good agreement with experimental observations. Moreover, it allows analysis of the HSR at different signal levels which are not easily accessible in experiments. An example is represented by our analysis of the double heat shock experiment, where it is interesting to note the (simulated) behaviour of e.g. SK* which is usually not measured in experiments (electronic supplementary material, figure S8).

We then investigated the system's response to a smooth and more natural variation in temperature mimicking a hot day (figure 4*a*–*f*). An interesting observation of the simulations was that the percentage of misfolded proteins does not exceed 0.25% of total proteins. While these numbers are not experimentally validated and should therefore be interpreted with care, it is remarkable that this fraction is a factor of 20 lower than the predicted 5% unfolded proteins, when a sharp temperature increase (of the same amount of degrees) is simulated (note the different scale of the vertical axis in figure 1*b*). Likewise, and possibly even more important, the maximal fraction of SK that is activated amounts to 80% upon a sharp temperature increase (figure 1*c*), while it reaches only approximately 4% for a smooth temperature variation (figure 4*g*). These results indicate that for the slow temperature increase the system is not really under stress, whereas the sharp temperature increase, usually applied in heat shock experiments, imposes severe stress, activating the vast majority of the available SKs.

We have finally studied how the maximal concentration of unfolded proteins reached during heat shock depends on the time *τ* of temperature increase from the minimal to the maximal value (figure 4*i*). This systematic investigation has shown that for times *τ* shorter than 1 min (i.e. a sharp increase in temperature) the maximal concentration of unfolded proteins is approximately 20 times higher than for increases that are slower than approximately 100 min. This result supports the notion that the HSR of *C. reinhardtii* is able to keep the concentration of unfolded proteins at negligible levels if the increase in temperature occurs on a timescale of several minutes to hours, in sharp contrast to the sudden increases usually applied in experiments. This indicates that the HSR of *C. reinhardtii* is indeed tailored to handle natural temperature variations due to a match of the intrinsic timescale of the HSR system, which could be easily tested in experiments. Moreover, as we have shown with several applications, our model provides a framework to explore further situations of interest and to generate new hypotheses to be tested by experiments.

Whereas models such as the one presented here may help greatly to elucidate molecular mechanisms and enable the interpretation of experimental data in a sound theoretical framework, they are clearly idealized and represent an ‘average’ cell. Thus, it is not straightforward to employ this model if one wishes to make predictions about the adaptive behaviour of a cell population, in particular, over longer timescales. However, in principle it seems possible that ensembles of models such as the one presented here, each with slightly modified parameters to reflect the plasticity found within a natural population, can be used to predict in which direction a population [36], or even whole ecosystems and their stoichiometries [37], may evolve under prolonged environmental perturbations.

## Data accessibility

The code implementing the model and the simulations in python can be freely downloaded from the following repository: https://github.com/QTB-HHU/ModelHeatShock.
